# Impact of genotyping (*PTPN2*, rs2542151) and (*MBOAT7*, rs641738) in prediction of fibrosis in Metabolic dysfunction- associated steatotic liver disease’ patients

**DOI:** 10.3389/fendo.2025.1615162

**Published:** 2025-09-11

**Authors:** Shimaa Abdelsattar, Hiba S. Al-Amodi, Hala F. M. Kamel, Zeinab A. Kasemy, Ehab Darwish, Asmaa Mosbeh, Ayman A. Sakr, Hanaa M. Elgazzar, Mervat Abdelkareem, Mai Abozeid, Shimaa K. Zewain, Hanan M. Bedair, Sabry M. Abdelmageed

**Affiliations:** ^1^ Clinical Biochemistry and Molecular Diagnostics Department, National Liver Institute, Menoufia University, Shebin El-Kom, Egypt; ^2^ Biochemistry Department, Faculty of Medicine, Umm Al-Qura University, Makkah, Saudi Arabia; ^3^ Medical Biochemistry and Molecular Biology Department, Faculty of Medicine, Ain Shams University, Cairo, Egypt; ^4^ Public Health and Community Medicine Department, Faculty of Medicine, Menoufia University, Shebin El-Kom, Egypt; ^5^ Faculty of Applied Health Sciences Technology, Menoufia National University, Tukh Tanbisha, Egypt; ^6^ Department of Hepatology, Gastroentrology and Infectious Diseases, Faculty of Medicine, Zagazig University, Zagazig, Egypt; ^7^ Department of Pathology, National Liver Institute, Menoufia University, Shebin El-Kom, Egypt; ^8^ Tropical Medicine Department, Faculty of Medicine, Menoufia University, Shebin El-Kom, Egypt; ^9^ Medical Microbiology and Immunology, National Liver Institute, Menoufia University, Shebin El-Kom, Egypt; ^10^ Hepatology and Gastroenterology Department, National Liver institute, Menoufia University, Shebin El-Kom, Egypt; ^11^ Internal Medicine Department, Faculty of Medicine, Menoufia University, Shebin El-Kom, Egypt, Shebin El-Kom, Egypt; ^12^ Clinical Pathology Department, National Liver Institute, Menoufia University, Shebin El-Kom, Egypt

**Keywords:** MASLD, genotyping, fibrosis, MBOAT7, rs641738, PTPN2, rs2542151

## Abstract

**Introduction:**

Numerous risk loci have been identified to have an essential role in Metabolic associated steatotic liver disease (MASLD) susceptibility and progression. The role of membrane-bound O-acyltransferase domain containing 7 (*MBOAT7*, rs641738) and protein tyrosine phosphatase non-receptor type 2 (*PTPN2*, rs2542151) genes in the risk of significant fibrosis in MASLD patients is still unclear. The aim of this study was to examine the association between *MBOAT7* rs641738 and *PTPN2* rs2542151 genotypes and the risk of significant fibrosis in Egyptian individuals with MASLD.

**Methods:**

We enrolled 142 patients with varying degrees of MASLD and 142 healthy controls with no evidence of MASLD. All subjects underwent biochemical tests and genotyping of *PTPN2* rs2542151 and *MBOAT7* rs641738 by real-time PCR. Additionally, patients were divided according to fibrosis stages assessed by transient elastography (Fibroscan) into 103 patients with early fibrosis (F0, F1) and 39 with significant fibrosis (≥ F2).

**Results and discussion:**

The study revealed that T allele and T/T genotype of *MBOAT7* rs641738 were more frequent among MASLD patients compared to controls, with higher frequency in the significant fibrosis subgroup compared to early fibrosis or control groups. Regarding *PTPN2* rs2542151, the G allele and G/G genotype were more frequent among MASLD patients compared to controls and showed higher frequency among the significant fibrosis group than controls. Multivariable regression analysis revealed that triglycerides, hepatic steatosis index, *MBOAT7* rs641738 (C/T+T/T), and *PTPN2* rs2542151 (G/T+G/G) were independent predictors of MASLD susceptibility. Only *PTPN2* rs2542151 (G/T+G/G) was the independent predictor of significant fibrosis in MASLD patients. In conclusion, *PTPN2* rs2542151 and *MBOAT7* rs641738 SNPs are associated with MASLD susceptibility, while only *PTPN2* rs2542151 mutations are associated with fibrosis progression.

## Introduction

Pathogenesis of metabolic dysfunction-associated steatotic liver disease (MASLD) is complex, thus its clinical presentation is varied. Due to the rising prevalence of obesity and metabolic syndrome, which are critical factors in the development and progression of the disease, it has emerged as the most prevalent liver disease globally, with a prevalence of about 30% (1). MASLD ranges from simple steatosis into nonalcoholic steatohepatitis, steatohepatitis with fibrosis, and cirrhosis (2).

Recently, the term of Non-alcoholic fatty liver disease (NAFLD) has been replaced with MASLD, as well as the term of non-alcoholic steatohepatitis (NASH) has been changed into metabolic dysfunction- associated steatohepatitis (MASH) in a worldwide agreement. A new diagnostic criteria based on the coexistence of steatosis and clinical evidence of obesity, hypertension with dysfunction in glucose, triglyceride (TG) and high density lipoprotien (HDL) metabolism has been also stated for both of them (3). However, several studies have displayed obvious over-lap between both defined patients with NAFLD and MASLD. (4–6).

The diagnosis necessitates the lack of extensive alcohol intake and other causes of hepatic fat accumulation, as well as the presence of imaging or histological evidence of hepatic steatosis. According to current guidelines, ultrasonography is the first-line imaging tool for diagnosing hepatic steatosis and is frequently performed to screen for MASLD (7). Patients also generally accept transient elastography as a painless, quick, and complication-free method of measuring liver stiffness. Currently, it’s advised as a rather reliable method for determining whether or not patients with MASLD have significant fibrosis (8). However, no well-performing tool is available for early prediction of MASLD; particularly, the levels of liver enzymes could be normal in those patients (9). Several studies investigated the risk factors and prediction risk scores for MASLD; however, their results are debated (10–12).

The fact that not all individuals with obesity develop MASLD and that disease rates vary throughout ethnic groups points to a genetic basis for MASLD (13, 14). The available data suggest that the transcription factor 4 (*TCM4*) gene is not highly expressed in the human liver (15). However, the available Expression Quantitative Trait Loci analysis suggests that rs641738 SNP present in the first exon of *TCM4* gene leads to C>T missense mutation, and leads to reduced expression and activity of the membrane bound O-acyltransferase domain containing 7 gene (*MBOAT7*), and perhaps participates in the progression of liver disease (15, 16). It was reported that sSNP; rs641738 is located a few hundred base pairs downstream of the 3′-untranslated region of *MBOAT7*, which belongs to a family of genes that encode specific acyl donors and acceptors (17) including lysophosphatidylinositol acyltransferase 1 (LPIAT1), which has a role in controlling the amount of free arachidonic acid in cells (18). Given its role in inflammatory lipid pathways, most mechanistic work relating to rs641738 has focussed on *MBOAT7* (19). Rs 641738, C>T is associated with lower hepatic expression of *MBOAT7* at both the mRNA (20) and protein levels (15). However, these findings have not been consistently replicated in different ethnicities (21) and there is a lack of data from the Middle East and African countries.

Protein tyrosine phosphatase non-receptor type 2 (PTPN2), formerly known as T-cell protein tyrosine phosphatase (TCPTP) due to its initial discovery in T cells, is another candidate gene identified by GWAS. It encodes the enzyme tyrosine-protein phosphatase non-receptor type 2, a member of the superfamily of protein tyrosine kinases (22). which is a dephosphorylation enzyme that can inhibit multiple inflammatory signaling pathways and regulate many biological processes as well as a variety of pathophysiological processes (23, 24). The intergenic SNP, rs2542151 is located in chromosome 18p11, 5.5 kb upstream of the *PTPN2* gene, showed the strongest association with Chrons disease (25, 26). The brain, liver, lung, and gastrointestinal system all have significant levels of *PTPN2* expression. Consequently, mutations in the *PTPN2* gene frequently lead to the development of inflammatory disorders such as Crohn’s disease, hepatitis, diabetes, and atherosclerosis (27, 28). Additionally, it was reported that SNPs in *PTPN2* rs2542151 in patients with MASLD was associated with higher severity of fatty liver disease and a higher prevalence of type 2 diabetes mellitus (T2DM) (29).

However, the MBOAT7 and *PTPN2* genes has been explored in several studies (15, 30–34), the relationship between the both genes and the risk of significant fibrosis in MASLD is still debatable due to a lack of evidence in different ethnicities especially in the Middle East and African countries. Therefore, we hypothesized to examine the association between *MBOAT7* rs641738 and *PTPN2* rs2542151 genotypes and the risk of significant fibrosis in Egyptian patients with MASLD.

## Subjects and methods

This case-control study included 142 patients with MASLD, who were recruited from the outpatient clinic of the National Liver Institute, Menoufia University, Egypt, and the endocrinology unit of the Internal Medicine Department, Faculty of Medicine, Menoufia University, Egypt. Exclusion criteria included age <18; patients with hepatic decompensation; other causes of chronic liver disease; autoimmune diseases; thyroid abnormalities; malignancy; sepsis; and patients consuming alcohol or receiving steatogenic drugs. In addition, 142 volunteers with no evidence of MASLD and matching age and sex were included as controls.

All subjects underwent full history-taking and physical examination. Waist circumference (WC) was measured at the top of the iliac crests using a non-stretchable tape. Body mass index (BMI) was calculated by devision of Weight in Kg by height squared.

### Sample collection and laboratory investigations

Eight mL of venous blood was withdrawn in the morning after an overnight fast. Two ml of blood was preserved on EDTA, to be used later for extraction of DNA. For CBC assay, one ml was evacuated into EDTA tube, CBC was assessed by the Sysmex XT-1800i automated hematology analyzer (Sysmex, Japan). Another 1 ml was used for INR assessment by The Sysmex CS-1600 Automated Hemostasis Testing (Sysmex Corporation, Kobe, Japan) and it was preserved on citrate. The remaining four ml was evacuated in a plain tube, centrifuged, and the resulting sera was divided into two aliquots: one for the assay of liver function tests as well as fasting blood sugar, lipid profiles, and creatinine using the Cobas e501 Auto analyzer (Roche-Germany), and the other for the insulin assay by the enzyme-linked immunosorbent assay (ELISA) method (Shanghai Sunred Biological Technology Co., Ltd. Catalogue No. 201-12-1720).

**Homeostasis model assessment (HOMA) index for insulin resistance:** calculated as [fasting insulin (µU/mL)×fasting glucose (mg/dL)/405] (35).

**Hepatic steatosis index:** calculated as [8 × ALT/AST + BMI + 2, if DM; +2, if female], with values < 30 ruling out and values > 36 ruling in steatosis (36).

**Conventional ultrasonography (US) examination:** performed for all subjects using the US system (iU22, Philips Medical Systems, Bothell, WA, USA) for the diagnosis of MASLD. Increased echogenicity in liver tissue relative to renal tissue is indicative of steatosis (37)^]^.

### Liver fibrosis assessment in MASLD patients

Liver stiffness measurement (LSM) was performed using transient elastography (Fibroscan, Echosens, France) through a right intercostal space with the patient in supine position, avoiding deep inspiration during breath hold, with the right arm extended. LSM above 7.1 kilopascal was defined for significant fibrosis (F ≥ 2) according to the manufacturer’s guidelines and previous studies (8). Patients were divided according to fibrosis stages into 103 patients with early fibrosis (F0, F1) and 39 with significant fibrosis (≥ F2).

### Gene polymorphism by real-time PCR

DNA was extracted from all samples using a spin column method according to the manufacturer’s instructions by Gene JET TM whole blood Genomic DNA Purification Mini Kit (Thermo Scientific, EU/Lithuania). Nanodrop spectrophotometer (UV spectrophotometer Q3000, Quawell Technology, Inc., USA) was used for determination of DNA concentrations.

*PTPN2* rs2542151 and *MBOAT7* rs641738 SNPs were analyzed utilizing the TaqMan SNP genotyping assay kit (Thermo Fisher Scientific, Waltham, MA, USA). Assay IDs for *PTPN2* rs2542151 and *MBOAT7* rs641738 were C_3043363_10 and C_8716820_10, respectively. Context Sequence [VIC/FAM] for rs2542151 and rs641738, respectively, were as follows: ACTTCGCCAATGCCTTGGTTCGGGC[G/T]CTTCCTGAGACTCTCATTTTCCTAA.

TCTGGCCTCCCGGGGGGCCAGCCAC[C/T]CCCTAGAGGAGCCCCAGGCTTCTGA.

The mixture of the PCR reaction consisted of 1 μL of genomic DNA (1–10 ng), 7.5 μL of TaqMan Genotyping Master Mixture (Applied Biosystems), 5.75 μL of nuclease-free water, and 0.75 μL of TaqMan SNP (probes). For the negative control reaction, 6 uL of DNAse-free water was added. PCR reactions for rs2542151 included enzyme activation at 95°C for 10 min, then 40 cycles were run at 92 °C for 15 seconds, and finally annealing at 60°C for 1 min. For rs641738, initial denaturation was at 95°C for 10 min, then 40 cycles were run at 95°C for 15 seconds (denaturing), followed by 60°C for 1 min (annealing/extension). PCR amplification was performed by a Rotor gene Q Real Time PCR System reaction (QIAGEN, GmbH-Germany). The results were analyzed with allelic discrimination software. If there was no nucleotide change in rs2542151 or rs641738 wildtype genotypes, G/G or C/C was assigned. If G changed to T at the rs2542151 SNP position, the T/T mutant genotype was assigned. If C changed to T at the rs641738 SNP position, the T/T mutant genotype was assigned. To ensure the accuracy and reliability of our genotyping results, we included duplicate samples in genotyping assays to verify the consistency and reproducibility of the results. The concordance rate between duplicate samples was >99%, indicating high genotyping accuracy. Additionally, we set a call rate threshold of >95% for both cases and controls, ensuring that only samples with high-quality genotyping data were included in the analysis.

### Sample size

The idea of an event per variable (EPV) of 20 is appropriate for logistic regression analysis, according to Austin and Steyerberg (38). Only independent variables with significant effect sizes are used in order to retain their results (39, 40). Consequently, an EPV of 8 was relevant, and 260 participants were required. After allowing for a 10% dropout rate, 288 participants were recruited. Six participants were excluded either due to refusal to participate (controls) or not fulfilling the study criteria (patients), leaving 284 participants (142 subjects per group).

### Statistical analysis

We used SPSS version 25.0 (SPSS Inc., Chicago, IL, USA), was used for statistical analysis. The independent t and ANOVA tests were used for parametric data. Kruskal-Wallis and Mann-Whitney tests were applied for non-parametric data. Pearson’s chi-square (χ2) test was used for comparing between two groups. Fisher’s exact test was employed in cases where at least one of the expected cells had a value below 5. A test of homogeneity of variances was performed. The Tukey test *post hoc* analysis was used for assumed equal variance, while the Dunnett T3 test was used for assumed unequal variance. Statistical significance was determined at a P value less than 0.05. To assess the effects of alleles and haplotypes, the 95% confidence interval (CI) and odds ratio (OR) were calculated. Each SNP was assessed for Hardy-Weinberg equilibrium (HWE) in patients with MASLD and controls to detect any deviations from expected genotype frequencies identifying potential genotyping errors or other issues that could impact the validity of our results. For additional analysis of the association between gene polymorphisms and risk of disease, OR was done in various genetic models (dominant, recessive, co-dominant 1, co-dominant 2, and overdominant). Multivariable binary logistic regression analysis was performed to detect the most associated predictors in relation to MASLD.

## Results

The BMI, waist circumference, diabetes mellitus, and hypertension were significantly higher in patients with MASLD compared to controls. The *PTPN2* rs2542151 genotype frequencies were matched with the HWE among controls and patients with MASLD. The *MBOAT7* rs641738 genotype frequencies were matched with the HWE among controls only, while it significantly differed from the HWE among patients with MASLD (**Table 1**). Hepatic steatosis index was significantly higher among the significant fibrosis subgroup than the early fibrosis or control groups. The laboratory characteristics of the studied groups were summarized in **Table 2**.

**Table 1:** Demographic, clinical characteristics and Hardy-Weinberg equilibrium of the studied groups(controls and total MASLD patients).

	Controls No = 142		Total MASLD patients No = 142		Test of sig		P value	
	Mean ± SD		Mean ± SD					
Age	41.6 ± 10.1		43.6 ± 11.0		t=1.60		0.111	
BMI (Kg/M^2^)	26.3 ± 1.1		29.7 ± 2.4		t=15.26		<0.001*	
Waist circumference (CM)	71.5 ± 3.6		90.4 ± 5.2		t=35.54		<0.001*	
	No	%	No	%				
Sex ▪ Female ▪ Male	79 63	55.6 44.4	72 70	50.7 49.3	χ^2^ = 0.69		0.405	
Hypertension	9	6.3	28	19.7	χ^2^ = 11.21		0.001*	
Diabetes Mellitus	14	9.9	55	38.7	χ^2^ = 32.18		<0.001*	
Hardy-Weinberg Equilibrium								
	Controls No = 142		χ^2^	P value	Total MASLD patients No = 142		χ^2^	P value
	Observed	Expected			Observed	Expected		
*MBOAT7* rs641738 ▪ C/C^®^ ▪ C/T ▪ T/T	81 48 13	77.6 54.7 9.6	2.14	0.143	30 44 68	19.0 65.9 57.0	15.69	<0.001*
*PTPN2* rs2542151 ▪ T/T^®^ ▪ G/T ▪ G/G	79 48 15	74.7 56.6 10.7	3.26	0.070	55 67 20	55.2 66.7 20.2	0.003	0.995

No, Number; BMI, Body mass index; t: independent t test; χ^2^: Pearson’s chi-square test; ^®^: Reference group; *Statistically significant at p < 0.05.

**Table 2:** Hepatic steatosis index and laboratory investigations of the studied groups.

	Controls No = 142	Early fibrosis No = 103	Significant fibrosis No = 39	P value	Total MASLD patients No = 142	P_4_
	Mean ± SD	Mean ± SD	Mean ± SD		Mean ± SD	
Hepatic steatosis index	36.4 ± 1.8	39.9 ± 3.0	42.7 ± 6.7	P_1,2,3_<0.001*	41.1 ± 4.5	<0.001*
Hemoglobin (gm/dl)	14.2 ± 1.2	13.3 ± 1.3	13.5 ± 1.1	P_1,2_<0.05*, P_3_>0.05	13.4 ± 1.2	<0.001*
WBCs ×10^3^/μL	7.0 ± 1.2	7.8 ± 1.5	7.1± 1.2	P_1,3_<0.05*, P_2_>0.05	7.6 ± 1.5	<0.001*
Platelets ×10^3^/μL	337.9 ± 45.3	307.2 ± 64.0	184.7 ± 16.6	P_1,2,3_<0.001*	273.6 ± 77.8	<0.001*
Albumin (g/dL)	4.6 ± 0.4	4.5 ± 0.4	3.9 ± 0.2	P_1_>0.05, P_2,3_<0.001*	4.4 ± 0.4	<0.001*
Billirubin (mg/dL)	0.6 ± 0.2	0.6 ± 0.2	0.7 ± 0.2	P_1_>0.05, P_2,3_<0.05*	0.6 ± 0.2	0.006*
INR	1.0 ± 0.02	1.0 ± 0.05	1.0 ± 0.03	P_1_>0.05, P_2,3_<0.05*	1.0 ± 0.04	0.009*
Total cholesterol (mmol/l)	155.4 ± 14.3	307.9 ± 52.6	355.9 ± 44.9	P_1,2,3_<0.001*	321.1 ± 54.9	<0.001*
HDL (mmol/l)	62.1 ± 8.8	55.7 ± 12.2	52.2 ± 5.9	P_1,2_<0.001*, P_3_>0.05	54.7 ± 10.9	<0.001*
LDL (mmol/l)	77.6 ± 11.9	228.8 ± 34.8	228.0 ± 24.1	P_1,2_<0.001*, P_3_>0.05	228.6 ± 32.2	<0.001*
Triglycerides (mmol/l)	114.7 ± 17.3	195.6 ± 49.5	200.1 ± 12.8	P_1,2_<0.001*, P_3_>0.05	196.8 ± 42.5	<0.001*
Creatinine (mg/dl)	0.6 ± 0.2	0.7 ± 0.2	0.8 ± 0.2	P_1,2_<0.01*, P_3_>0.05	0.7 ± 0.2	<0.001*
	Median (IQR)	Median (IQR)	Median (IQR)		Median (IQR)	
Fasting insulin (mmol/l)	10.1 (8.9-11.3)	30 (25-120)	170 (39-200)	P_1,2,3_<0.001*	31 (25-154.5)	<0.001*
FBG (mmol/l)	4.6 (4.3-5.1)	6.1 (5.4-8.1)	10.9 (6.7-18.5)	P_1,2,3_<0.001*	6.6 (5.5-10.2)	<0.001*
HbA1c (%)	4.6 (4.4-4.9)	4.9 (4.6-6.9)	7.9 (5.9-9.3)	P_1,2,3_<0.001*	5.2 (4.8-7.9)	<0.001*
HOMA-IR	2.1 (1.8-2.4)	7.4 (5.9-42.9)	87.9 (11.4-155.9)	P_1,2,3_<0.001*	8.6 (6.2-69.9)	<0.001*
AST (U/L)	23 (17-30)	39(27-55)	102 (89-139)	P_1,2,3_<0.001*	51 (29.8-80.3)	<0.001*
ALT (U/L)	26 (19-33)	51(37-77)	61 (52-76)	P_1, 2_<0.001*, P_3_<0.05*	57 (39-76.3)	<0.001*

No, Number; SD, Standard deviation; WBCs, White blood cells; INR, International normalized ratio; HDL, High-density lipoprotein; LDL, Low-density lipoprotein; FBG, Fasting blood glucose; HbA1c, Hemoglobin A1C; HOMA-IR, Homeostasis model assessment for Insulin resistance; AST, Aspartate transaminase; ALT, Alanine transaminase; IQR, Interquartile range; Statistical tests, t test and Mann-Whitney for 2 groups, ANOVA and Kruskal-Wallis for > 2 groups; P_1_, Controls vs. Early fibrosis, P_2_, Controls vs. Significant fibrosis; P_3_, Early vs. Significant fibrosis; P_4_, Controls vs. Total patients; *Statistically significant at p < 0.05.

**Table 3** illustrates the genotype and allele distribution of *MBOAT7* rs641738 and *PTPN2* rs2542151 polymorphisms among MASLD patients and controls, further stratified by fibrosis stages. Notably, the T/T genotype of *MBOAT7* rs641738 was significantly more frequent in MASLD patients compared to controls (47.9% vs. 9.2%), with an OR of 14.12 (95% CI: 6.83–29.20, p < 0.001), and this association was even stronger in the significant fibrosis subgroup (OR = 68.54, 95% CI: 18.33–256.32, p < 0.001). For *PTPN2* rs2542151, although the G/G genotype showed only a trend toward association with MASLD overall (OR = 1.92, 95% CI: 0.90–4.07, p = 0.078), its frequency was markedly elevated in patients with significant fibrosis (OR = 21.07, 95% CI: 5.30–83.77, p < 0.001), suggesting a specific role in fibrosis progression.

**Table 3:** *MBOAT7* rs641738 and *PTPN2* rs2542151 genotypes and alleles among the studied groups.

	Controls No = 142		Total MASLD patients No = 142		χ^2^	P value	OR (LL – UL 95% CI)
	No	%	No	%			
*MBOAT7* rs641738							
▪ C/C^®^ ▪ C/T ▪ T/T	81 48 13	57.0 33.8 9.2	30 44 68	21.1 31.0 47.9	- 8.39 60.72	- 0.002* <0.001*	1.0 2.48 (1.38-4.44) 14.12 (6.83-29.20)
▪ **C** ^®^ ▪ **T**	210 74	73.9 26.1	104 180	36.6 83.4	80.02	<0.001*	1.0 4.91 (3.43-7.03)
*PTPN2* rs2542151							
▪ T/T^®^ ▪ G/T ▪ G/G	79 48 15	55.6 33.8 10.6	55 67 20	38.7 47.2 14.1	- 7.34 2.91	- 0.006* 0.078	1.0 2.0 (1.21-3.32) 1.92 (0.90-4.07)
▪ **T** ^®^ ▪ **G**	206 78	72.5 27.5	177 107	62.3 37.7	6.74	0.009*	1.0 1.60 (1.12-2.28)
	Controls No = 142		Early fibrosis No = 103		χ^2^	P value	OR (LL – UL 95% CI)
*MBOAT7* rs641738							
▪ C/C^®^ ▪ C/T ▪ T/T	81 48 13	57.0 33.8 9.2	27 41 35	26.2 39.8 34.0	- 9.58 31.86	- 0.001* <0.001*	1.0 2.56 (1.40-4.68) 8.08 (3.73-17.47)
▪ **C** ^®^ ▪ **T**	210 74	73.9 26.1	95 111	46.1 53.9	39.34	<0.001*	1.0 3.32 (2.26-4.85)
*PTPN2* rs2542151							
▪ T/T^®^ ▪ G/T ▪ G/G	79 48 15	55.6 33.8 10.6	52 43 8	50.5 41.7 7.8	- 1.25 0.20	- 0.262 0.655	1.0 1.36 (0.79-2.34) 0.81 (0.32-2.05)
▪ **T** ^®^ ▪ **G**	206 78	72.5 27.5	147 59	28.6 71.4	0.08	0.774	1.0 1.06 (0.71-1.58)
	Controls No = 142		Significant fibrosis No = 39		χ^2^	P value	OR (LL – UL 95% CI)
*MBOAT7* rs641738							
• C/C^®^ ▪ C/T ▪ T/T	81 48 13	57.0 33.8 9.2	3 3 33	7.7 7.7 84.6	- 0.40 68.98	- 0.672 <0.001*	1.0 1.69 (0.33-8.70) 68.54 (18.33-256.32)
▪ **C** ^®^ ▪ **T**	210 74	73.9 26.1	9 69	11.5 88.5	99.72	<0.001*	1.0 21.76 (10.34-45.76)
*PTPN2* rs2542151							
▪ T/T^®^ ▪ G/T ▪ G/G	79 48 15	55.6 33.8 10.6	3 24 12	7.7 61.5 30.8	- 23.35 28.47	- <0.001* <0.001*	1.0 13.17 (3.76-46.08) 21.07 (5.30-83.77)
▪ **T** ^®^ ▪ **G**	206 78	72.5 27.5	30 48	38.5 61.5	31.31	<0.001*	1.0 4.23 (2.50-7.15)

No, Number; χ2, Pearson’s chi-square test; ^®^, Reference group; OR, odds ratio; CI, confidence interval; *Statistically significant at p < 0.05.

**Table 4** further confirms these findings through multiple genetic models, demonstrating that the dominant model for *MBOAT7* rs641738 (C/T+T/T vs. C/C) conferred an approximately fivefold increased risk of MASLD (OR = 4.96, 95% CI: 2.94–8.36, p < 0.001), with even higher risk in the recessive (OR = 9.12, 95% CI: 4.72–17.62, p < 0.001) and co-dominant models. Meanwhile, the *PTPN2* rs2542151 dominant model (G/T+G/G vs. T/T) showed a significant, albeit moderate, association with MASLD susceptibility (OR = 1.98, 95% CI: 1.24–3.18, p = 0.004). Interestingly, the overdominant model revealed a protective effect of the G/T heterozygote (OR = 0.57, 95% CI: 0.35–0.92, p = 0.021), indicating a potential heterozygote advantage in disease risk.

**Table 4:** Comparison between the control and total patients’ groups according to *MBOAT7* rs641738 and *PTPN2* rs2542151 gene polymorphisms in different genetic models.

	OR (LL – UL 95% CI)	P value
*MBOAT7* rs641738		
C/C^®^ vs. C/T+T/T (Dominant)	4.96 (2.94-8.36)	<0.001*
C/C+C/T^®^ vs. T/T (Recessive)	9.12 (4.72-17.62)	<0.001*
C/C^®^ vs. C/T (Co–dominant–1)	2.48 (1.38-4.44)	0.002*
C/C^®^ vs. T/T (Co–dominant–2)	14.12 (6.83-29.20)	<0.001*
C/T^®^ vs. C/C+T/T (Over dominant)	1.14 (0.69-1.87)	0.612
*PTPN2* rs2542151		
T/T^®^ vs. G/T+G/G (Dominant)	1.98 (1.24-3.18)	0.004*
T/T+G/T^®^ vs. G/G (Recessive)	1.39 (0.68-2.83)	0.366
T/T^®^ vs. G/T (Co–dominant–1)	2.0 (1.21-3.32)	0.006*
T/T^®^ vs. G/G (Co–dominant–2)	1.92 (0.90-4.07)	0.087
G/T^®^ vs. T/T+G/G (Over dominant)	0.57 (0.35-0.92)	0.021*

OR, Odds ratio; CI, Confidence interval; *Statistically significant; ^®^, Reference group.

The BMI, fasting insulin, FBG, HbA1c, HOMA-IR, AST, and fibroscan score were significantly higher among patients with MASLD having C/T+T/T genotypes of *MBOAT7* rs641738 than those having C/C genotype. For *PTPN2* rs2542151, the BMI, total cholesterol, fasting insulin, FBG, HbA1c, HOMA-IR, AST, ALT, and fibroscan score were significantly higher among patients with MASLD with G/T+T/T genotype than those with T/T genotype (**Table 5**).

**Table 5:** Distribution of different parameters according to *MBOAT7* rs641738 and *PTPN2* rs2542151 genotypes among MASLD patients.

	*MBOAT7* rs641738		P value	*PTPN2* rs2542151		P value
	C/C No = 30	C/T+T/T No = 112		T/T No = 55	G/T+G/G No = 87	
	Mean ± SD	Mean ± SD		Mean ± SD	Mean ± SD	
Hepatic steatosis index	41.2 ± 4.1	40.5 ± 4.6	0.425	39.9 ± 3.6	41.1 ± 4.9	0.130
BMI(Kg/M^2^)	28.7 ± 1.8	29.9 ± 2.5	0.005*	28.6 ± 1.9	30.3 ± 2.5	<0.001*
Total cholesterol	313.7 ± 50.8	323.1 ± 55.9	0.544	309.7 ± 53.6	328.2 ± 54.6	0.050*
**HDL**	54.0 ± 14.5	54.9 ± 9.8	0.861	56.6 ± 13.3	53.6 ± 8.9	0.155
**LDL**	232.8 ± 33.0	227.4 ± 31.9	0.346	231.5 ± 36.5	226.6 ± 29.1	0.382
Triglycerides	195.3 ± 48.0	197.3 ± 41.0	0.586	196.6 ± 54.6	196.9 ± 32.8	0.971
	Median (IQR)	Median (IQR)		Median (IQR)	Median (IQR)	
Fasting insulin	26.2 (25-35)	39 (25.1-170)	0.013*	26 (25-35)	130 (26-180)	<0.001*
FBG	5.4 (5-6.2)	6.7 (5.8-10.5)	<0.001*	5.6 (5.3-6.4)	8.6 (6.1-11.1)	<0.001*
HbA1C %	4.8 (4.5-5)	5.9 (4.9-8.6)	<0.001*	4.8 (4.5-5.2)	6.8 (5.0-9.2)	<0.001*
HOMA-IR	6.3 (5.7-8.4)	11.2 (6.5-79.3)	0.001*	6.5 (5.9-8.8)	46.1 (6.8-93.7)	<0.001*
AST	37 (25-55)	55.5 (32.5-94)	0.004*	31 (25-54)	68 (41-102)	<0.001*
ALT	54 (36.5-70.5)	57.5 (42-77)	0.256	48 (36-72)	61 (48-77)	0.021*
Fibroscan score	4.3 (3.5-5.2)	6.3 (5.2-8.7)	<0.001*	4.9 (4-5.5)	7.5 (5.7-9.5)	<0.001*

No, Number; SD, Standard deviation; HDL, High-density lipoprotein; LDL, Low-density lipoprotein; FBG, Fasting blood glucose; HbA1c, Hemoglobin A1C; HOMA-IR, Homeostasis model assessment-Insulin resistance; AST, Aspartate transaminase; ALT, Alanine transaminase; IQR, Interquartile range; *Statistically significant at p < 0.05 written in bold.

**Table 6** presents multivariable logistic regression analyses adjusted for clinical variables and confirms that both *MBOAT7* rs641738 (C/T+T/T) and *PTPN2* rs2542151 (G/T+G/G) genotypes independently predict MASLD susceptibility with striking odds ratios of 17.02 (95% CI: 9.80–295.66, p = 0.005) and 8.88 (95% CI: 5.06–155.84, p = 0.010), respectively. In addition to genetic factors, triglycerides (OR = 1.15, 95% CI: 1.06–1.25, p = 0.001) and hepatic steatosis index (OR = 7.89, 95% CI: 2.07–30.02, p = 0.002) were significant independent clinical predictors. Importantly, regarding the prediction of significant fibrosis, *PTPN2* rs2542151 (G/T+G/G) was the only independent genetic predictor with a notably high OR of 23.36 (95% CI: 2.87–189.73, p = 0.003), whereas the MBOAT7 variant and traditional biochemical markers (AST, ALT) did not maintain significance in the adjusted model. These results emphasize the robust predictive value of these polymorphisms, particularly *PTPN2* rs2542151, for MASLD risk stratification and fibrosis progression in the Egyptian population studied.

**Table 6:** Multivariable logistic regression analysis for predictors among the studied groups.

MASLD susceptibility	P value	OR	95% CI	
			Lower	Upper
Triglycerides	0.001*	1.15	1.06	1.25
Hepatic steatosis index	0.002*	7.89	2.07	30.02
*MBOAT7* rs641738 (C/T+T/T)	0.005*	17.02	9.80	295.66
*PTPN2* rs2542151(G/T+G/G)	0.010*	8.88	5.06	155.84
AST	0.123	1.19	0.95	1.44
Hypertension	0.403	4.93	0.11	20.78
ALT	0.222	1.11	0.93	1.33
Diabetes Mellitus	0.816	1.47	0.05	38.97
Risk of significant fibrosis				
*PTPN2* rs2542151(G/T+G/G)	0.003*	23.36	2.87	189.73
ALT	0.327	1.01	0.99	1.03
Triglycerides	0.450	1.0	0.99	1.02
*MBOAT7* rs641738 (C/T+T/T)	0.354	2.97	0.29	29.92

OR, Odds ratio; CI, Confidence interval; *Statistically significant.

## Discussion

MASLD is a complicated illness where the environment and susceptibility genes combine to affect the disease’s severity (41). Population-based research in multi-ethnic cohorts have demonstrated significant inter-ethnic diversity in susceptibility to MASLD. African-Americans exhibit a diminished propensity for developing MASLD relative to Europeans, but Asians, especially Hispanics, face an elevated risk (42). The inter-ethnic disparities were not explained by type 2 diabetes, obesity, or socioeconomic variables (43). However, uncertainty surrounds the genetic components linked to MASLD pathogenesis. GWAS has identified a variety of SNPs linked to hepatic steatosis and fibrosis, some of which are less reliably replicated and seem to be affected by ethnicity (44, 45).

Dietary factors and inherited variants in genes that play important roles in antioxidant defense, such as glutathione S-transferase Mu 1 (GSTM1), glutathione S-transferase theta 1 (GSTT1), cytochrome P450 superfamily members, and sulfotransferase 1A1 (SULT1A1), have been found to interact significantly with high fruit intake (more than two fruits/day) or high consumption of grilled meat/fish (more than once per week). This increases the risk of developing MASLD and may be related to the steatosis caused by aromatic hydrocarbons, as severe MASLD is characterized by oxidative stress and mitochondrial dysfunction (46).

Indeed, energy intake has increased generally in the Egyptian population’s nutritional pattern during the last 50 years. Nutrition shifted toward a diet with reduced consumption of fresh fruits and vegetables while increasing consumption of processed foods, fast food, red meat, vegetable oils, and soft beverages (47). According to estimates, up to 40% of the fat that Egyptian women consume is saturated fat (48), and up to 80% of them consume insufficient amounts of fresh fruit and vegetables each day. Similarly, the average prevalence of inadequate physical activity in Egypt is greater (31.0%) than the global average (27.5%), according to Guthold et al. (49), Egypt is one of the top 10 nations in the world for obesity rates (49). Also according to specific studies, the prevalence range of MASLD in Egypt is roughly 47.5%, with 56.7% having fibrosis (50), so the nature of liver disease in Egypt is changing from one of communicable to noncommunicable diseases (51, 52).

Liver fibrosis is the key prognostic factor in patients with MASLD (53). Currently, there is insufficient evidence to establish a robust connection between *MBOAT7* rs641738 and *PTPN2* rs2542151 gene polymorphisms and fibrosis progression in MASLD, particularly in the Middle East and African countries. Therefore, we investigated the potential role of these genotypes in the prediction of significant fibrosis in Egyptian patients with MASLD.

Our study showed that *MBOAT7* rs641738 T allele and T/T genotype were more frequent among patients with MASLD than controls, with higher frequency in the significant fibrosis subgroup than the early fibrosis or control groups. *In vitro* and *in vivo* research indicates that hepatic *MBOAT7* downregulation induces *de novo* lipogenesis, triglyceride synthesis, and hepatic lipid accumulation (54). It can promote liver inflammation and fibrosis by altering lipid composition and triggering the release of cytokines and fibrogenic mediators (55). Recent studies indicated that the *MBOAT7* risk mutation was linked to hepatic fibrosis regardless of inflammation, indicating that hepatocyte signaling in fibrogenic mesenchymal cells can induce fibrosis (56, 57). Notably, MBOAT7 has been shown to be one of the single nucleotide polymorphisms that are strongly linked to the onset of MASLD and the advancement of the disease. Understanding the biology of these genetic variations has led to new discoveries in the fields of lipid droplet remodeling, hepatic very low-density lipoprotein secretion, and lipogenesis (41).

Several studies revealed that *MBOAT7* rs641738 is implicated in several hepatic diseases, involving alcohol-related cirrhosis and liver fibrosis in chronic hepatitis B and C, as well as hepatocellular carcinoma (HCC) (18, 33, 58, 59). However, other studies didn’t reveal any association with liver disease progression or HCC development (60–64).

In patients with MASLD, Mancina et al. initially reported, irrespective of obesity, a link between the rs641738 variants and elevated hepatic fat content, liver damage, and an increased risk of increased necroinflammation and fibrosis in individuals of European origin (15). This may be because of the association of this variant with necro-inflammation, but with no hepatocellular ballooning. Predominantly, *MBOAT7* variant was found to be independently associated with fibrosis’ development, which represents the major determinant for the diagnosis of patients with MASLD (65, 66) suggesting that a common etiology for these conditions is related to alteration of hepatic lipid metabolism. (15).

Subsequently, conflicting findings about the association between MASLD and the rs641738 variant have been published. Xia et al.’s meta-analysis showed no relation of *MBOAT7* rs641738 with the risk of MASLD (67). A different meta-analysis reported that the rs641738 C>T variant is a risk factor for the presence and severity of MASLD in Caucasians (21). The discrepancy among different ethnic groups implies that genetic and environmental factors interact to determine the susceptibility and severity of MASLD and liver fibrosis. Furthermore, the T allele exhibits significant variability among populations, with an allelic frequency of 0.37 in the global population. In fact, the frequency of this minor allele in the 1000 Genomes project varies from 0.44 for European to 0.32 for African and 0.22 for Asian ancestry (68). A systematic review concluded that the published evidence supports the association between *MBOAT7* rs641738 C>T and increased MASLD susceptibility and severity as well as risk of advanced fibrosis in subjects with MASLD from Caucasian, Hispanic, and African American ethnicities, with contradictory findings in most studies on Asian populations (69).

More recently, this genetic variant was not found to be significantly associated with MASLD in the Indian population (70) or Korean subjects with lean MASLD (71), while in Chinese patients, it was associated with increased MASLD occurrence but not related to fibrosis in two studies (72, 73), additionally,it was found to promote inflammation and fibrosis in another study (74), however it was not related to the risk of MASLD in a different study (75). As previously mentioned, studies reported an association between *MBOAT7* rs641738 and MASLD in Europeans; however, two recent studies found no association in Mexican-origin individuals (76) and Caucasian subjects from Romania (77), suggesting a potential genetic variation within ethnic sub-populations.

In the Middle East and African countries, data on the relation between *MBOAT7* rs641738 and MASLD is lacking. To the far of our knowledge, this study might be the first to assess the relation between *MBOAT7* SNPs and significant fibrosis in Egyptian patients with MASLD. A study involving Egyptian patients with HCV-related liver fibrosis revealed a significant correlation between the *MBOAT7* T/T genotype and advanced fibrosis (78). In our study, BMI, fasting insulin, FBG, HbA1C, and HOMA-IR were significantly higher among C/T+T/T than C/C genotype. The rs641738 T allele has been associated with lower hepatic expression of *MBOAT7* at both the mRNA and protein levels (68). *MBOAT7* knockdown in the liver and adipose tissue of mice promoted hepatic steatosis and inflammation, hyperinsulinemia, and insulin resistance (79). We found no relation between ALT and the C/T+T/T variant. In the meta-analysis by Teo et al., the rs641738 C>T variant showed no effect on insulin resistance and a positive relation with ALT in Caucasians but not in non-Caucasian populations (21). A Mendelian randomization analysis pointed to a causal role of genetically determined steatosis in the determination of insulin resistance mediated by the degree of liver damage (80). We also found that AST and fibroscan scores were higher among C/T+T/T genotype, denoting a potential role of the T allele in fibrosis progression. However, on multivariable regression analysis, the C/T+T/T genotype was an independent predictor of MASLD susceptibility but didn’t predict significant fibrosis.

We also examined the role of *PTPN2* rs2542151 genotypes in MASLD. The G allele and G/G genotype of the *PTPN2* rs2542151 were more frequent among patients with MASLD compared to controls. Also, the G/T and G/G genotypes were higher among patients with significant fibrosis. Furthermore, the GT+GG genotype was the only predictor of significant fibrosis. In line with our results, Miele et al. recently demonstrated that the *PTPN2* rs2542151 T>G variant is associated with the severity of fibrosis in Caucasian patients with MASLD (29).

PTPN2 is an intracellular enzyme encoded by the *PTPN2* gene. It has an important role in negatively regulating various immunological pathways through the dephosphorylation of various signaling proteins. It has a significant role in the inflammatory signaling for several immune cells and intestinal epithelial cells (81). Loss of PTPN2 in intestinal epithelial cells promotes the secretion of inflammatory cytokines and dysfunction of the intestinal barrier, which are vital factors in MASLD pathogenesis. It leads to disruption of intracellular junction proteins, increased intestinal permeability, disturbance of gut microbiome, and promotes the translocation of microbes into blood circulation, which has an essential role in the development of liver steatosis and fibrosis progression (82). GWAS revealed that loss-of-function mutations in *PTPN2* were associated with increased intestinal permeability, which is an early etiological event of chronic immune diseases, such as inflammatory bowel disease and celiac disease (83).

We found that the rs2542151 G/T+G/G genotype was associated with significantly higher BMI, fasting insulin, FBG, HbA1C, and HOMA-IR than the T/T genotype. Consistently, Miele et al. found that the GT/GG genotype was independently associated with diabetes (29). PTPN2 has been reported to have a role in glucose metabolism. It regulates signal transduction of insulin by inactivation of its receptor through dephosphorylation mechanisms of the β-chain. In the liver, PTPN2 deficiency results in enhancement of the signaling of growth hormone, insulin resistance, increased weight and hepatic steatosis (84). Also, G/T+G/G genotype was associated with significantly higher ALT, AST, and fibroscan scores in our study, pointing to the role of the rs2542151 mutation in MASLD severity and fibrosis progression. Recently, partial *PTPN2* deletion in dendritic cells was found to be associated with liver inflammation (85). In the study by Miele et al., no difference was noted in the distribution of genotypes between MASLD patients with high AST and ALT and those without high transaminases (29).

When we performed multivariable logistic regression analysis, the *MBOAT7* rs641738 C/T+T/T and the *PTPN2* rs2542151 G/T+G/G genotypes, as well as serum triglycerides and hepatic steatosis index, were the independent predictors of MASLD susceptibility. This confirms the possible role of these genetic variants in the pathogenesis of hepatic steatosis. High serum triglyceride level has been reported as a key factor in the development of MASLD and is an important marker for predicting MASLD even in lean patients (86). The Hepatic Steatosis Index (HSI) has shown excellent diagnostic performance in previous studies and has been suggested as a useful tool for predicting MASLD (87). The only independent predictor of significant fibrosis in our study was the *PTPN2* rs2542151 G/T+G/G genotype, which strengthens a proposed pathogenetic hypothesis of *PTPN2* rs2542151 as a predisposing factor of impaired gut permeability that correlates with the severity of MASLD (29). Notably, PTPN2 treatment significantly decrease serum TG, total cholesterol, and LDL levels, as well as reducing metabolic disturbances and hyperglycemia in mice (88). Additionally, taken the theatrical role of PTPN2 and CD8+ T cells in MASH pathogenesis, therapeutic targeting of PTPN2 might importantly enhance outcomes for patients with MASLD as well as MASH. (89).

The genetic differences seen among genetically distinct ethnic groups may then be a result of genomic evolution and selection (nutritional genomics). The in-depth understanding of diet-genome interactions may enable the use of novel nutritional and lifestyle strategies for the prevention and management of chronic illnesses through precision nutrition, which may be a component of customized medicine therapy (90) improving the results of MASLD and associated comorbidities (91).

## Study limitations

Our study has several limitations that should be considered when interpreting the results: The cross-sectional nature of our study limits our ability to infer temporal relationships between genotypes and fibrosis progression. Longitudinal studies are needed to further explore these dynamics. The use of hospital-based volunteers as controls may introduce selection bias, potentially affecting the generalizability of our findings. Future studies could benefit from community-based recruitment strategies. The reliance on ultrasound for diagnosing the absence of MASLD in controls may not detect mild steatosis due to its limited sensitivity. More precise diagnostic methods might be necessary for accurate classification.

In conclusion, our study identified the *PTPN2* rs2542151 G/T+G/G genotype as an independent predictor of significant fibrosis among Egyptian patients with MASLD. Furthermore, the *MBOAT7* rs641738 C/T+T/T and *PTPN2* rs2542151 G/T+G/G genotypes were significantly associated with increased risk and susceptibility to MASLD. These findings hold potential implications for genetic screening and personalized risk stratification in MASLD patients. However, validation in prospective cohorts is necessary to confirm these results and elucidate their clinical utility. Future research should prioritize larger, multi-regional studies to further investigate the role of these genetic variants in MASLD pathogenesis and progression.

## Data Availability

The datasets presented in this study can be found in online repositories. The names of the repository/repositories and accession number(s) can be found below: https://www.ncbi.nlm.nih.gov/gene/5771/ and https://www.ncbi.nlm.nih.gov/gene/79143/.
